# Determining the Intersection of Social Prescribing in Social Work Practice: Protocol for a Scoping Review

**DOI:** 10.2196/75235

**Published:** 2025-08-29

**Authors:** Rachelle Ashcroft, Simon Lam, Tin D Vo, Keith Adamson, Benjamin Walsh, Rebecca Bliss, Stefaniia Martsynkevych, Matthew R Langiano, Shaista Okhai

**Affiliations:** 1 Factor-Inwentash Faculty of Social Work University of Toronto Toronto Canada; 2 John P. Robarts Research Library University of Toronto Toronto, ON Canada; 3 Hamilton Family Health Team Hamilton, ON Canada; 4 Department of Social Work McMaster University Hamilton Canada

**Keywords:** social prescription, social conditions, systems navigation, systematic review, social work

## Abstract

**Background:**

Social prescribing is a nonclinical intervention used in various health care settings to improve health outcomes of individuals by attending to the social determinants of health and broader social factors. Prevalence of social prescribing has increased globally over the past decade, leading to the creation of new organizations and networks dedicated to social prescription. Although social workers comprise one of the largest providers of social and mental health services sectors, there remains little guidance how social workers can integrate social prescribing in practice.

**Objective:**

The objectives of this scoping review are 3-fold. The objectives of this scoping review are to (1) systematically scope the literature on social prescribing and social work and identify scholarly gaps in the literature, (2) identify the role of social work in social prescribing, and (3) describe how social workers are integrating and engaging in social prescribing in clinical practice.

**Methods:**

The review follows the 5-stage scoping review framework from Arksey and O’Malley (2005), which was later enhanced by Levac et al in 2010. The review will examine both academic and grey literature. We will search for studies in the following databases: MEDLINE, Embase, PsycINFO, CINAHL, Social Services Abstracts, and Social Work Abstracts. Grey literature will be searched using Google with a focus on social prescription organizations, social prescription conferences, and Canadian social prescription reports. All studies must be in English and there are no date restrictions. Title and abstract screening, assessment of full-text review, and data extraction will be conducted by 2 independent reviewers. Data will be extracted into a chart format, which will be analyzed for data summarization and synthesis.

**Results:**

The results of the study and submission of a manuscript for peer review are expected in October 2025. The results of the scoping review are expected to contribute to an understanding of how social workers employed in health care can integrate social prescribing in their practice.

**Conclusions:**

To the authors’ knowledge, this is the first scoping review undertaken on the topic of social prescribing and social work. Findings from the scoping review will inform the future development of guidelines to support the integration of social prescription in social work practice.

**International Registered Report Identifier (IRRID):**

DERR1-10.2196/75235

## Introduction

The field of social prescribing has gained momentum over the last decade, with social prescribing becoming increasingly prevalent across more than 20 countries, including Canada, the United States, Australia, the United Kingdom, and elsewhere [1,2]. There has been a rise of new social prescribing organizations and networks established across the globe [2], such as the World Health Organization– and United Nations–linked Global Social Prescribing Alliance [1], the Social Prescribing Network [3], the National Academy for Social Prescribing [4], and the Canadian Institute for Social Prescribing [5]. Originating in the United Kingdom, social prescribing refers to a process of care that connects patients to nonmedical services and enhances health and well-being [6,7]. According to the Social Prescribing Network, social prescribing is defined as “enabling health care professionals to refer patients to a link worker, to co-design a nonclinical social prescription to improve their health and well-being” [8]. Various definitions of social prescribing exist; however, one commonality across different definitions is that social prescribing is considered a nonclinical intervention [2,9]. Although the Social Prescribing Network and the World Health Organization situates social prescribing within a health care setting involving a health care provider [8,10], Muhl et al [2] hold a more expansive view and assert that social prescribing can occur in nonmedical community settings. Inherently, the essence of social prescribing is attendance to the social determinants of health, health equity, and the broad health-related social factors that influence health outcomes [2,7]. This aligns well with social work, which takes a holistic approach by looking at how history, the environment, social structures, and other factors impact the health of individuals.

Social work is a practice-based profession that responds to the needs of individuals, families, and communities by focusing on improving health and social well-being [11,12]. As one of the largest groups of health and social service professionals [11,13], social workers have a long-standing history of practice in a broad range of health settings, such as hospitals, primary health care, community mental health, public health, social services, nonprofit organizations, and elsewhere [14]. Notably, health was one of the earliest areas of practice for social work and continues to be one of its largest practice domains [15,16]. Guided by foundational values of social justice, human rights, respect for diversity, and collective responsibilities, social work inherently strives for health equity and alleviation of existing and future disparities [14,17-19]. The person-in-environment perspective guides social workers to bring a crucial understanding of the disparities and inequities that manifest as health and mental health conditions, with practice aimed at addressing the range of nonmedical psychosocial factors that impact health and well-being [14]. Social workers fulfill many roles with crisis management, service coordination, case management, problem solving, discharge planning, systems navigation, resource allocation, and community mobilization as core functions [16,17,20]. Furthermore, social workers often engage in referrals to community resources to attend to the socioeconomic factors impacting the health of individuals [21]. This is aligned to social prescribing, which recognizes the importance of nonmedical services that are often beyond the scope of health care professionals [7]. At the same time, there are no standards for how social prescribing occurs or who is involved throughout the process of social prescription [7]. Despite the similarities in focus, it is unclear how social work and social prescription are related or intersect to inform one another. From a health systems design perspective, having clarity about the roles and functions of similar types of services and providers can “optimise human well-being and overall system performance” [22].

The objectives of this scoping review are to (1) systematically scope the literature on social prescribing and social work and identify scholarly gaps in the literature, (2) identify the role of social work in social prescribing, and (3) describe how social workers are integrating and engaging in social prescribing in clinical practice. This work will constitute the first step in the development of guidelines to explain how social prescription and social work are aligned. By doing so, this information will help provide guidance to social workers and other leaders in health care in determining how social work practice can best integrate social prescription in practice and how health care organizations who are implementing social prescription can use social workers for this function. Furthermore, the knowledge gained will inform future research directions that can also shift clinical practice and policy.

## Methods

### Protocol Design

Using scoping review methods, our study is helping to provide a broader understanding of social work’s engagement in social prescription. A scoping review is a method of knowledge synthesis that maps key concepts and evidence related to a defined field of research by systematically searching and synthesising existing knowledge [23]. Systematically mapping a subject field is particularly useful when minimal literature exists for a particular topic or when the examination is on a complex or nonhomogeneous topic [23-25]. Scoping reviews provide an opportunity to identify key concepts and evidence that can help guide clinical practice [24]. Knowledge synthesis, including scoping reviews, is necessary for advancing health care practices and can help knowledge users—in this case social workers—increase integration of efficient evidence-based decisions in clinical practice [23]. The study adheres to the PRISMA-ScR (Preferred Reporting Items for Systematic reviews and Meta-Analyses extension for Scoping Reviews) checklist, and no protocol was registered. In alignment with scoping review designs, no quality appraisal will be performed on included sources.

Following recommendations from Colquhoun et al [23], our methods for this study are based on a 5-stage scoping review framework proposed by Arksey and O’Malley [24] and enhanced by Levac et al [26]. The five stages informing our review are (1) identifying the research question, (2) identifying relevant studies, (3) selecting studies, (4) charting the data, and (5) summarizing data and synthesizing results [24,26]. We consider this an appropriate framework for our current study due to the infancy of the topic and scarcity of evidence-based studies.

### Stage 1: Identifying the Research Question

Levac et al [26] recommend clarifying stage 1 [24] by combining a broad research question with a clear scope of inquiry that defines the concept and target population to clarify the focus of the scoping review. Levac et al [26] also recommend developing the research question with the intended outcome of the scoping review in mind to help determine the purpose of the study. In this case, the purpose of this scoping review is to provide clarity about the ways that social work has been involved in social prescribing that may contribute to clinical practice guidelines that are currently absent. Through consultation, the research team has defined the overarching research question as, “In what ways has social work been involved with social prescribing?” In addition, the two subquestions guiding this scoping review are as follows: (1) “How has social work contributed to the role of social prescribing?” and (2) “How are social workers integrating and engaging in social prescribing in clinical practice?”

### Stage 2: Identifying Relevant Studies

In stage 2 [24] we seek to identify available literature on social prescription that includes mention of social work. Levac et al [26] recommend strengthening stage 2 by assembling a suitable research team with combined content and methodological expertise to ensure successful completion of the scoping review. We have followed this suggestion and have assembled a team that combines expertise in social prescribing (RA, KA, TDV, and SO), social work practice in health care (RA, KA, TDV, RB, SM, and MRL), and scoping review methodology (RA, SL, KA, TDV, and BW). We have also identified 2 graduate-level research assistants who will participate in all phases of the scoping review (SM and MRL).

A search strategy will be implemented to identify literature discussing both social prescription and social work. This strategy, developed by a social sciences librarian (BW) and peer-reviewed by an independent health science librarian using the Peer Review of Electronic Search Strategies framework [27] for use in MEDLINE (Ovid) will then be translated for use in 5 additional databases including CINAHL, Social Work Abstracts, Social Services Abstracts, Applied Social Sciences Index and Abstracts, and Scopus. The goal of this search will be to locate all articles in the social work and health science literatures with relevant text words appearing in the title, abstract, and author-supplied keyword fields. Relevant controlled vocabulary will also be used to increase search sensitivity. All text words and subject headings have been determined collaboratively with the research team. No restrictions on publication date, language, or geographical location were applied to ensure the comprehensive capture of relevant literature. The pilot MEDLINE search is shown in Table 1.

**Table 1:** 

Search statement	Results
(social work* or socialwork*).tw,kf.	21,074
exp Social Work/ or Social Workers/	20,346
(social work* or socialwork*).tw,kf. OR exp Social Work/ or Social Workers/	33,861
(prescrip* or prescrib*).tw,kf.	317,451
(social work* or socialwork*).tw,kf. OR exp Social Work/ or Social Workers/ AND (prescrip* or prescrib*).tw,kf.	469
link work*.tw,kf.	246
community connector*.tw,kf.	24
link work*.tw,kf. OR community connector*.tw,kf.	269
(social work* or socialwork*).tw,kf. OR exp Social Work/ or Social Workers/ AND (prescrip* or prescrib*).tw,kf. OR link work*.tw,kf. OR community connector*.tw,kf.	715

A search in each database was conducted, and results were exported to the literature review management software Covidence [28] for deduplication and screening. While a total of 10,416 citations were exported from 6 databases, deduplication left 2543 unique sources to be screened. Full search strategies across all 6 databases are available on request.

In addition, a grey literature search will be conducted to identify any relevant reports using the following steps: (1) review of websites belonging to any agencies or associations mentioned in articles included in the sample; (2) review of websites belonging to national and international social prescription associations; (3) review of any conferences, symposiums, or gatherings specific to social prescription; and (4) review of all grey literature in Canada that is focused on social prescription. The grey literature search will capture reports created by professional associations and health care organizations pertaining to social prescription and social work.

Terms will be searched as keywords in the titles, abstracts, and subject headings as appropriate. Search results will be downloaded and imported into the web-based platform Covidence [28].

### Stage 3: Selecting Studies

In stage 3 [24], the review process will be comprised of two levels of screening: (1) a title and abstract review, and (2) a full text review. The stage 3 inclusion criteria guiding publication types acceptable for review are broad and inclusive of all peer-reviewed publications such as original research, case reports, literature reviews, technical guidelines, and commentary papers. Only articles published in the English language will be included at this stage. No date limits will be applied, and no limits on geography will be applied.

For the first level of screening, the 2 graduate-level research assistants (SM and ML)—working under supervision of the lead author (RA) and the project coordinator (SL)—will independently conduct title scans and abstract reviews to assess eligibility against the inclusion criteria. Disagreements regarding the inclusion of articles in the title and abstract reviews will be decided by the lead investigator or project coordination. Articles that are considered relevant by both research assistants will be included in the full text review. During full text screening, both research assistants will assess each article to determine eligibility. Any discordant full text articles will be reviewed by both the research assistants and the lead investigator or project coordinator to determine whether they meet the inclusion or exclusion criteria. A hand search of reference lists of the articles included in the sample will be conducted and included in our screening process.

Relevant studies will be assessed against the following inclusion criteria: (1) the words social prescription (inclusive of search terms) and social work (inclusive of search terms) are used in the title or abstract and (2) social prescription is a key focus of the article. Any type of study design will be included, as well as commentary articles. We will follow the recommendation by Levac et al [26] to approach stage 3 as an iterative process that includes regular team meetings to discuss the criteria for study inclusion and exclusion throughout the study process. Screening will occur using Covidence [28].

### Stage 4: Charting the Data

To guide stage 4 [24], a data collection instrument will be generated by the research team to extract characteristics from the sample. We will extract data from all studies included in the scoping review. Example of categories that will be included on the data charting form include but are not limited to authorship, publication year, type of article (eg, original study or commentary paper), study design (qualitative, quantitative, or mixed), geographical origin of study or article, definition of social prescription, social prescription activities, description of practice setting (community-based, hospital, primary care, or web-based), description of social work activities related to social prescribing, type of practice location, and patient population characteristics. This form, created in Microsoft Excel, will be created with input by the research team as per the recommendation by Levac et al [26] that the research team collectively determine variables to extract from the data and collaboratively develop the data charting form to effectively answer the research questions. Data extraction will be conducted by the 2 graduate-level research assistants working under the supervision of the lead investigator and project coordinator.

### Stage 5: Summarizing Data and Synthesizing Results

The focus of stage 5 [24] will be to provide a synthesis and summary of the results. An inductive qualitative content analysis is generally descriptive in nature and appropriate to guide analysis of this scoping review data [29]. Following data charting, the findings from the selected sources will be synthesized, with an emphasis on identifying recurring themes relevant to the objectives of the review. This aligns with the aims of scoping reviews to provide a map of concepts underpinning the research, key sources, and types of research [30]. Levac et al [26] suggest breaking stage 5 into the following three smaller steps: (1) analysis, (2) reporting of the results pertaining to the overall study purpose and research question, and (3) consideration of the meaning of the results and discussion of the potential implications that the findings may have on future research, clinical practice, and policy.

### Ethics and Dissemination

This study will be the first step toward developing guidelines for social workers pertaining to social prescription. Research ethics approval is not required given that we are collecting data from publicly available sources. The results of this scoping review study will be disseminated through a conference presentation that engages an audience of social work practitioners in health care and a peer-reviewed publication. Most members of the research team have established relationships with social work and health care networks, which will also be used to disseminate the findings. Our aim is to use the results from this scoping review to guide future research on social prescription with social workers.

## Results

The scoping review commenced in February 2025. The formal literature search was completed in February 2025 with the exception of the addition of “community connectors,” which was added to the formal literature search in July 2025. The process of screening all articles was completed in March 2025 with the exception of articles identified with the updated term “community connectors,” which was completed in July 2025. We are currently in the process of conducting the grey literature search. The results of the study and submission of a manuscript for peer review are expected in October 2025. The results of the scoping review are expected to contribute to an understanding of how social workers employed in health care can integrate social prescribing in their practice. Gaps in social work practice and social prescribing may also emerge.

## Discussion

### Overview

This scoping review addresses an important gap in understanding the role of social workers in social prescribing. Although a core focus of social workers’ roles in various settings is on addressing the social determinants of health [14,31,32], such as issues related to financial challenges and insecure housing, research to inform social workers’ uptake of social prescribing in practice appears minimal. While advances in social prescribing have been made, current literature aimed at informing and guiding social workers in the practice of social prescribing appears absent. Given social workers’ historical expertise in addressing the social dimensions of health, providing mental health care, working with complex client situations, and doing systems navigation and resource allocation, there is much that the discipline of social work can add to the social prescribing literature and practice. This scoping review intends to identify the role of social work in social prescribing and explain how social workers can integrate social prescribing in clinical practice.

Given the exploratory nature of the research questions and the anticipated variability in study designs, populations, and outcomes, a scoping review represents a well-suited methodological approach for this topic. The use of gray literature will supplement the search to find pertinent information not found in the databases and will include literature that may be guiding social prescribing in various health and community settings. This scoping review will constitute an important first step in the development of practice guidelines for social workers and social prescribing. The findings will assist leaders in health care in determining how to allocate social work resources pertaining to social prescribing.

### Limitations

The scoping review has some limitation. First, the search is limited to sources available in English, meaning that sources not written in English will not be included, thus limiting generalizability. Although acceptable within the context of scoping reviews, studies included in the sample will not be evaluated for quality or methodological rigor.

### Conclusions

The scoping review will assist in the further development and implementation of social prescribing by providing a foundation by which to develop guidelines for social work practice. These results will be disseminated at a national social work conference and will be published in a health care journal. This scoping review aims to address a gap in the literature and provide guidance on the implementation of social prescribing practices in health care.

## Abbreviations

PRISMA-ScR: Preferred Reporting Items for Systematic reviews and Meta-Analyses extension for Scoping Reviews

## Data Availability

The datasets generated or analyzed during this study will be made available in the final manuscript. All data will be attained from publicly available sources.
